# Synoptic taxonomy of *Cortaderia* Stapf (Danthonioideae, Poaceae)

**DOI:** 10.3897/phytokeys.76.10808

**Published:** 2017-01-11

**Authors:** Daniel Testoni, H. Peter Linder

**Affiliations:** 1 Herbario BBB, Departamento de Biología, Bioquímica y Farmacia, Universidad Nacional del Sur, San Juan 670, CP-8000 Bahía Blanca, Argentina; 2 Department of Systematic and Evolutionary Botany, University of Zurich, Zollikerstrasse 107, CH-8008 Zurich, Switzerland

**Keywords:** Leaf anatomy, key, nomenclature, South America, taxonomy

## Abstract

*Cortaderia* (Poaceae; Danthonioideae) is a medium-sized genus of C3 tussock grasses, widespread in the temperate to tropic-alpine regions of South America. It is particularly important in the subalpine and alpine zones of the Andes. We revised the classification of the genus, and recognize 17 species grouped into five informal groups. We describe one new species, *Cortaderia echinata* H.P.Linder, from Peru. We provide a key to the groups and the species, complete nomenclature for each species including new lectotypes, and notes on the ecology, distribution and diagnostic morphological and anatomical characters.

## Introduction

*Cortaderia* Stapf (Danthonioideae, Poaceae) is best known for the pampas-grass, *Cortaderia selloana* (Schult. & Schult. f.) Asch. & Graebn., which is globally cultivated as a garden ornamental (Grounds 1979), but which is also an aggressive invader in many warm-temperate regions (Gadgil et al. 1990; Harradine 1991; Lambrinos 2001; Okada et al. 2007; Robinson 1984). However, the genus is also a major component of the temperate C3 grasslands of South America, from Tierra del Fuego in the south to Venezuela in the north, and from the Atlantic coastal mountains near Rio de Janeiro to the Equadorian Andes, and from sea level at the southern extreme to over 4500 m at the equator.

The genus was erected by Stapf (1897), initially to include only the large tussocks allied to *Cortaderia selloana*, which until then had been classified under *Gynerium* Bonpl. In the first decades of the 20^th^ century Pilger (1906) and Hackel (in Dusén 1909) transferred four species from *Gynerium* into *Cortaderia*. During the second half of the last century five new species were described by Swallen (1948; 1956). Recently, Linder et al. (2010) segregated five New Zealand species into *Austroderia* N.P.Barker & H.P.Linder and a New Guinean species into *Chimaerochloa* H. P. Linder on the basis of morphology and molecular data, and included *Lamprothyrsus* Pilg., a genus of two species distributed in South America, in *Cortaderia*.

The taxonomy of the genus has never been reviewed in total, from a global perspective. The *Cortaderia selloana* group was revised by Acevedo de Vargas (1959) and Testoni and Villamil (2014), and *Lamprothyrsus* by Conert (1961), Bernardello (1979) and Testoni (2016). In addition, there have been numerous regional treatments in diverse South American floras, including Ecuador, Peru and Bolivia (Hitchcock 1927), Patagonia (Nicora 1978), Peru (Tovar 1993), Argentina (Astegiano et al. 1995), Ecuador (Laegaard 1997), Bolivia (Renvoize 1998) and Venezuela (Davidse 2004).

There are several taxonomic problems in the genus. Species delimitations of the three species closely related to *Cortaderia selloana* present a major challenge, as already noted by Stapf (1897). These have recently been revised by Testoni and Villamil (2014), based on field and herbarium studies. Similar species delimitation problems are also evident in some of the Andean tussock species. There are several very local species, apparently known only from the type localities – the validity of these species could be questioned. As well, there are several apparent segregates from currently accepted species; these may be due to “over-splitting” of taxa. Finally, in 2008 Paul Peterson and Rob Soreng collected a putatively new species of *Cortaderia* from Peru, which needs a formal description.

The reproductive biology of *Cortaderia* is complex, with apparently hermaphrodite, dioecious, gynodioecious and apomictic species. Connor (1965) showed that gynodioecy, rather than dioecy, is the common and widespread condition in *Cortaderia*. The variation in reproductive structure in the genus was explored in detail in Connor (1973). He showed several syndromes. Only *Cortaderia sericantha* (Steud.) Hitchc. was monomorphic, with the reproductive organs in the male and bisexual plants scarcely differentiated. Testoni and Villamil (2014) established that species of the Selloana group (Sect. Cortaderia sensu Conert 1961) are gynodioecious and apomictic (e.g. *Cortaderia selloana* can form extensive clonal populations, one of which gave rise to Cortaderia selloana subsp. jubata (Lemoine) Testoni & Villamil) or only apomictic species (e.g. *Cortaderia speciosa* (Nees & Meyen) Stapf), whereas the species in the rest of the genus are dimorphic and dioecious, except for *Cortaderia hieronymi* (Kuntze) N.P.Barker & H.P.Linder, which Connor and Dawson (1993) showed to be apomictic. The genetics of the gynodioecism was explored for *Cortaderia selloana* by Connor and Charlesworth (1989), who showed that it was probably controlled by a male-sterility gene, expressed in the female-only plants of the species. The reproductive system influences the patterns of variation in the species, and might account for the taxonomic complexity of some species.

The phylogeny of *Cortaderia* is as yet incompletely known. Phylogenetic analyses have been published by Barker et al. (2003) and Pirie et al. (2008), based on which the New Zealand and New Guinean species were separated into the genera *Austroderia* and *Chimaerochloa*, whereas the South American *Lamprothyrsus* was included in the genus *Cortaderia* (Linder et al. 2010). *Cortaderia* in its current circumscription is monophyletic, and most closely related to the “danthonioid” clade of the Danthonioideae (Pirie et al. 2009).

This paper presents a critical review of the species limits in *Cortaderia* based on leaf anatomical features, investigation of field populations and the study of herbarium specimens. We also clarify the nomenclature and typification of all names in the genus, and provide a key to the species. A descriptive monograph of the whole subfamily is in preparation, and full descriptions will be published in that account, as well as the full lists of specimens examined.

## Materials and methods

The morphological descriptions were compiled from the analysis of the available herbarium material at B, BA, BBB, CONC, CORD, K, LOJA, M, NY, P, RB, SI, SGO, QCA, US, W, Z and ZT (acronyms follow Thiers (continuously updated)). Where sufficient material was available, spikelets were dissected, sketched and measured. The lemmas were mounted in glycerine, and drawn using a camera lucida. Anatomical investigation was based on leaf fragments of herbarium specimens (Table 1). Fragments ca. 1 cm long, from near the middle of blade, were first softened and reconstituted in warm, soapy water for 20–40 min. Transverse sections were hand-cut at 20–40 µm, and epidermal scrapes were prepared of the abaxial surface. Sections and scrapes were differentially stained with a combination of Safranin Red and Alcian Blue (Tolivia and Tolivia 1987), dehydrated in an alcohol series, and mounted in Histomount. In addition, for *Cortaderia selloana*, *Cortaderia speciosa* and *Cortaderia vaginata* samples were fixed in formalin-acetic acid-alcohol, dehydrated in an ethyl alcohol-tertiary butyl alcohol series and embedded in Paramat. The study plant sections (20 μm) were stained with safranin-fast green and mounted in Canada balsam. The anatomy of each species was described using the characters and character states proposed by Ellis (1976; 1979).

**Table 1.** Vouchers for anatomy.

Species	Voucher	Country
*araucana*	Testoni, D., 656 (BBB)	Chile
*araucana*	Werdermann, E., 1360 (K)	Chile
*bifida*	Beck, S.G., 1816 (US)	Bolivia
*bifida*	Renvoize, S.A.; Cope, T.A.; Beck, S., 4202 (K)	Bolivia
*bifida*	Smith, D.N. & Canabilla, J., 7167 (US)	Peru
*bifida*	Testoni, D., 477 (BBB)	Ecuador
*bolivensis*	Beck, S.G., 21266 (K)	Bolivia
*boliviensis*	Beck, S.G., 11273 (K)	Bolivia
*boliviensis*	Renvoize, S.A., 5342 (SI)	Bolivia
*columbiana*	Schultes, R.E., 7251 (K)	Venezuela
*columbiana*	Schultes, R.E., 7226 (K)	Colombia
*columbiana*	Schulz, J.P. & Rodri, L., 318 (US)	Venezuela
*egmontiana*	Green, S.W., 42385 (K)	Falkland/Malvinas
*egmontiana*	Moore, D.M., 1697 (K)	Argentina
*egmontiana*	Peterson, P.M., Soreng, R.J. & Refulio-Rodriguez, N., 17465 (US)	Argentina
*egmontiana*	Pisano, E. & Henriquez, M., 8802 (CONC)	Chile
*egmontiana*	Testoni, D., 634 (BBB)	Argentina
*echinata*	Peterson, P.M. & Soreng, R.J., 21587 (Z)	Peru
*hapalotricha*	Laegaard, S., 53805 (K)	Ecuador
*hapalotricha*	Renvoize, S.A. & Laegaard, S., 5023 (K)	Peru
*hieronymi*	Asplund, E. 11971 (K)	Peru
*hieronymi*	Garcia, Beck, S.G. & Michel 563 (K)	Bolivia
*hieronymi*	Testoni, D., 386 (BBB)	Argentina
*hieronymi*	Testoni, D., 496 (BBB)	Ecuador
*modesta*	Carauta, P., 927 (RB)	Brazil
*modesta*	Chase, A., 8288 (US)	Brazil
*modesta*	Glaziou, A.F., 17913 (K)	Brazil
*modesta*	Luetzelburg, 6368 (M)	Brazil
*nitida*	Laegaard, S., 53121 (K)	Ecuador
*nitida*	Testoni, D. 516 (BBB)	Ecuador
*nitida*	Soderstrom, T.R., 1350 (K)	Colombia
*nitida*	Steyermark, J.& Dunsterville. G.C.K., 101134 (US)	unknown
*roraimensis*	Farney, C. 885 (RB)	Brazil
*roraimensis*	Magire, B., Pires, J.M. & Magire, C.K., 60448 (US)	Venezuela
*roraimensis*	Steyermark, J., 103836 (US)	Venezuela
*speciosa*	Renvoize, S.A., 5341 (K)	Bolivia
selloana ssp. selloana	Linder, H.P., s.n.	South Africa
selloana ssp. selloana	Villamil, CB., 11738 (BBB)	Uruguay
selloana ssp. jubata	Testoni, D., 435 (BBB)	Ecuador
*sericantha*	Laegaard, S., 55066 (K)	Ecuador
*sericantha*	Laegaard, S., 55728 (P)	Ecuador
*sericantha*	Ramsay, P.M.; Merrow-Smith, P.J., 967 (K)	Ecuador
*sericantha*	Testoni, D., 438 (BBB)	Ecuador
*speciosa*	Renvoize, S.A.; Flores, G.; Peca, C., 5272 (K)	Bolivia
*speciosa*	Testoni, D., 644 (BBB)	Chile
*vaginata*	Reitz, P.R., 2672 (US)	Brazil
*vaginata*	Smith, L.B., Reitz, P.R. & Klein, R., 7761 (B)	Brazil
*vaginata*	Zanin 1654 (BBB, FLOR)	Brazil

The assignation of holo- and lectotype status follows the analysis and recommendations of (McNeill 2014).

## Taxonomic treatment

### Cortaderia

Taxon classificationPlantaePoalesPoaceae

Stapf, Gard. Chron. ser. 3. 22: 378 (1897) nom. cons.

Cortaderia Stapf, Gard. Chron. ser. 3. 22: 378 (1897) nom. cons. Type species: Cortaderia selloana (Schult.) Asch. & Graebn. (Syn. Mitteleur. Fl. 2(1): 325. 1900) (Basionym Arundo selloana Schult.).
Moorea Lem., Ill. Hort. 2: Misc. 14 (1855) nom. rej., non Rolfe (1890). Type species: Moorea argentea (Nees) Lem. (Cortaderia selloana).
Lamprothyrsus Pilg., *Bot. Jahrb. Syst.* 37 (Beibl. 85): 58 (1906). Type species: Lamprothyrsus hieronymi (Kuntze) Pilg. (Basionym Triraphis hieronymi Kuntze).

#### Description.

**Figure 1.**
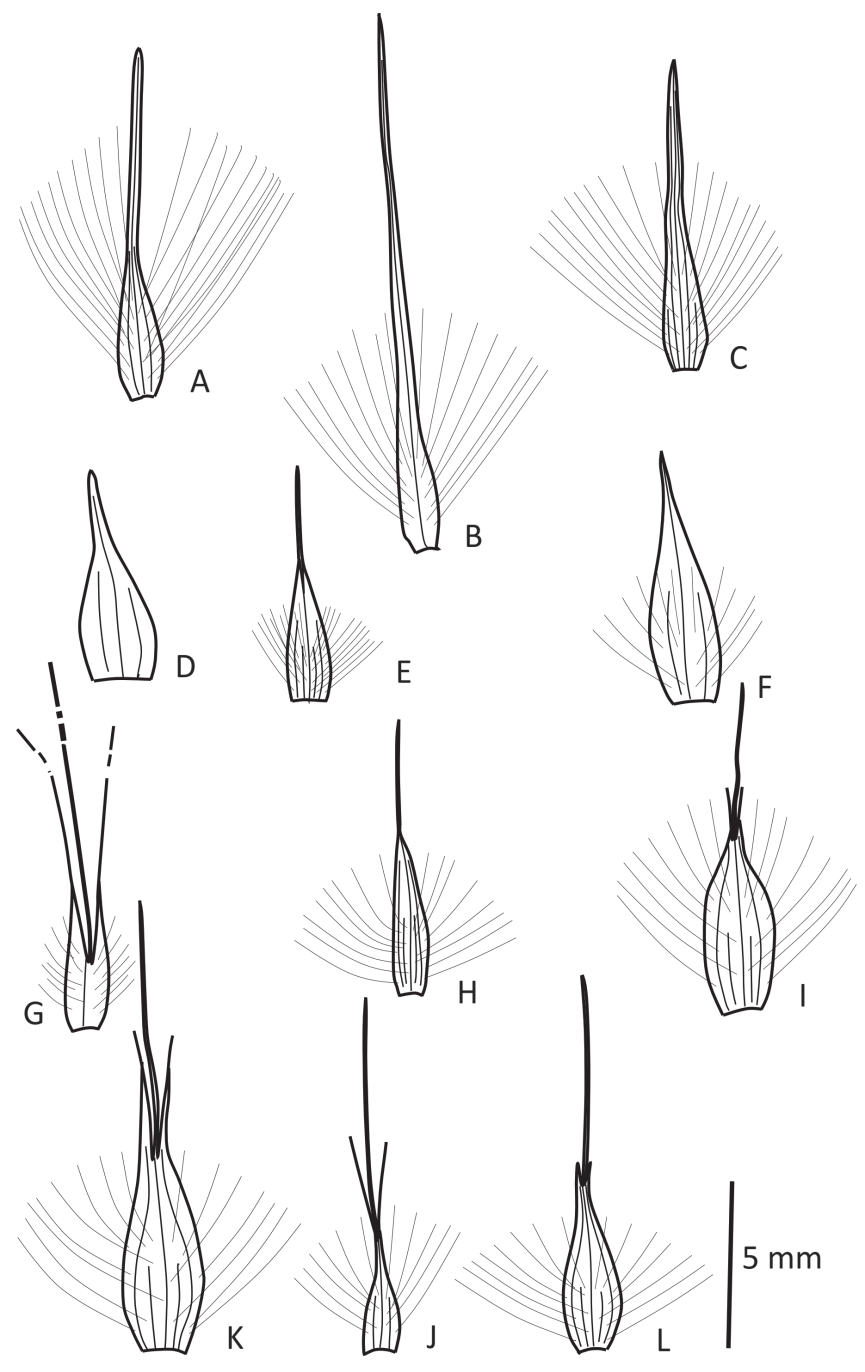
Lemmas of selected species of *Cortaderia*. **A** *Cortaderia selloana*, *Jürgens 40* (B) **B** *Cortaderia araucana*, *Borchers* s.n. (US) **C** *Cortaderia speciosa*, *P.M. Peterson 12766* (US) **D** *Cortaderia vaginata*, *L.B. Smith, P.R. Reitz & R. Klein 7761* (B) **E** *Cortaderia egmontiana*, *P.M. Peterson, R.J. Soreng & N. Refulio-Rodriguez 17508* (US) **F** *Cortaderia modesta*, *A. Chase 8288* (US) **G** *Cortaderia hieronymi*, *A. Burkart et al. 30395* (P) – note that awn and setae are much longer than illustrated **H** *Cortaderia nitida*, *S. Laegaard 52786* (K) **I** *Cortaderia sericantha*, *E. Asplund 17175* (B) **J** *Cortaderia bifida*, *D.N. Smith & J. Cabanillas 7167* (US) **K** *Cortaderia hapalotricha*, *J.C. Solomon & R. Chevalier 16620* **L** *Cortaderia columbiana*, *J.P. Schulz 318* (US). All at same magnification.

Gynodioecious, dioecious, hermaphrodite or apomictic perennials, ranging from rounded vegetable hedgehogs less than 0.5 m tall to erect 4 m tall tussocks; innovations intravaginal; spreading stolons rare. Leaf sheaths variable: persisting intact, or fragmenting transversely, or decaying into a tangled mass of fibres, or occasionally persisting as burnt-off sheaths; glabrous or more rarely covered in a dense indumentum. Ligule of one or many rows of cilia, to 5 mm long. Leaf blades to 2 m long, tough, expanded, rolled or folded, occasionally pungent, usually persistent but occasionally disarticulating above the ligule, sometimes with an adaxial weft of hairs directly above the ligule; margins sometimes roughly scabrid and cutting. Inflorescences paniculate, sometimes compact but usually plumose, to 1 m long, many-spikeleted, pedicels and pulvini glabrous, scabrid or villous. Spikelets to 30 mm long, with 2–10 florets, disarticulating above the glumes, male spikelets usually less hairy than female spikelets and glabrous in the Selloana group; glumes glabrous, often papery or membranous, 4–30 mm long, usually 1-veined and rarely with no veins, upper and lower glumes similar. Lemmas (Fig. 1) 3–7 nerved, mostly with the central three nerves continuing into a more or less twisted awn; the lateral nerves sometimes terminating in lateral bristles, the lemmas often continuing up the awns, consequently with the bristles apparently borne on the awn, in *Cortaderia selloana* the lemma continues to the tip of the awn and so obscures the awn; lemmas usually long-villous on the back, rarely glabrous. Palea membranous, linear, often longer than the lemma, keeled, sometimes variously villous on the back. Lodicules two. Anthers three, fertile or sterile, to 3.5 mm long. Ovary stalked, styles two. Caryopses 1.5–3.5 mm long, variable in shape, glabrous, embryo mark from ¼ to more than ½ length of caryopsis, hilum linear, from ¼ to ¾ caryopsis length.

#### Leaf anatomy.

Leaf in transverse section sclerophyllous, leaves varying from expanded to setaceous, margins not thickened but with a sclerenchyma cap. Adaxial furrows vary from deep and cleft-like to absent; abaxial ribs sometimes present. Vascular bundles differentiated into two, rarely three, orders; primary vascular bundles 6–30, symmetrically distributed in the two leaf sections; either ad- or abaxially or centrally positioned, circular or elliptical, sometimes with sclerosed phloem; outer bundle sheath cells always distinct from the chlorenchyma and sometimes lignified, entire or interrupted by bundle sheath; adaxial sclerenchyma as narrow girders, as trapezoidal girders, as T-shaped girders or inversely anchor-shaped girders; abaxial sclerenchyma as small strands, as narrow girders, as wide girders, as trapezoidal girders, or as massive linked girders forming a continuous subepidermal layer; tertiary vascular bundles 1-several between the primary vascular bundles, adaxial sclerenchyma as small strands, as narrow girders, as trapezoid girders narrowing towards vascular bundles, as T-shaped girders or inversely anchor-shaped girders; abaxial sclerenchyma absent, as small strands, as narrow girders, as broad girders, as trapezoidal girders or as massive linked girders forming a continuous subepidermal layer. Mesophyll of small, angular isodiametric chlorenchyma cells with small air spaces; mesophyll islands of colourless cells usually absent, sometimes with colourless collenchyma cells connecting the adaxial and abaxial furrows and so partitioning the chlorenchyma. Abaxial subepidermal layer sometimes with collenchymatous or non-chlorophyllous cells in 1-several layers only along the margins, or flanking the midrib, and sometimes with this layer extending over the whole width of the leaf. Abaxial epidermal zonation present or absent; microhairs or macrohairs absent; silica bodies absent, or tall and narrow, or round and single. Adaxial epidermis sometimes with papillae, prickle-hairs, and microhairs.

#### Distribution and ecology.

Widespread in South America, from Tierra del Fuego (Argentina) to Venezuela, from Brazil to Peru, from sea level to the Páramo.

### Systematics

We arranged the species into five informal groups, which are coherent morphologically and anatomically.

#### Key to the species (anatomical characters in brackets)

**Table:** 

1	Lemma body continued up the awn, for at least the same length as the expanded portion of the lemma; plants forming massive tussocks to 4 m tall, inflorescences plumose (leaves with abaxial groves (Fig. 2a–c))	**Selloana group...2**
–	Lemma body not continued up the awn, lemmas consequently acute or obtuse or lobed, usually obviously awned; plants and inflorescences various (leaves rarely with abaxial grooves)	**5**
2	Glumes 9–17 mm long, ca. ½ length of basal lemmas; basal lemmas 14–25(–30) mm long; plants of southern (austral) Andean region	**2. *Cortaderia araucana***
–	Glumes 5–14 mm long, almost as long as or longer than the basal lemmas; basal lemmas 6–15 mm long; plants from southern Brazil, Uruguay, and Argentina northwards to Colombia	**3**
3	Lemma awn present above the insertion of the lateral setae (these often lost on herbarium material); spikelets 8–15 mm long; lemmas 7.0–12.5 mm long; glumes 6–8 mm long; plants from desert regions of the Andes	**3. *Cortaderia speciosa***
–	Lemma awn absent; spikelets 10–20 mm long; lemmas 6–15 mm long; glumes 5–14 mm long; widely distributed in South America	**4**
4	Gynodioecious plants, exceptionally populations exclusively pistillate; panicles pyramidal to fusiform, dense to lax, included or not in the foliage; southern Brazil, Uruguay and Argentina	**1a. Cortaderia selloana subsp. selloana**
–	Only pistillate plants; panicles pyramidal, lax, much exserted above the foliage; northwest Argentina to Colombia	**1b. Cortaderia selloana subsp. jubata**
5	Glumes without veins; lemmas with awns 14–35 mm long; sheaths always intact (primary vascular bundles with lignified sheaths and girders, tertiary vascular bundle sheaths and girders collenchyma)	**Lamprothyrsus Group: 5. *Cortaderia hieronymi***
–	Glumes with 1 (rarely 2) vein; lemmas awnless or with awns up to 17 mm long; when longer than 13 mm the basal sheaths are lacerated, sheaths and girders of all vascular bundles similar	**6**
6	Lemmas acute, at most with vestigial lobes, mostly without awns; from southern and eastern South America (leaves with large bulliform cells – Fig. 3A–C)	**Egmontiana group...7**
–	Lemmas lobed, often with setae on the lobes, mostly with awns; from the Andes and the tepui (bulliform cells absent or poorly developed)	**9**
7	Inflorescence compact with the branches shorter than the spikelets; leaf blades disarticulating from a persistent sheath; southern South America (leaf anatomy with adaxial ribs, phloem-pole usually intact)	**5. *Cortaderia egmontiana***
–	Inflorescences plumose with the branches longer than the spikelets; leaf blades persistent on the sheath; eastern Brazil (leaf anatomy with hardly any ad- or abaxial grooves and with the phloem-pole split)	**8**
8	Glumes 8–12 mm long; lemma back villous; basal sheaths burnt off, ensheathing the tiller bases	**6. *Cortaderia modesta***
–	Glumes 4–6 mm long; lemma back glabrous; basal sheaths breaking up into fibres	**7. *Cortaderia vaginata***
9	Old leaf sheaths intact, or shattering transversally, rarely some lacerated (sometimes in *Cortaderia boliviensis*); (leaves, except in *Cortaderia echinata*, with a multilayered wide collenchyma below the adaxial epidermis and no sclerenchyma girder connecting the vascular bundle to the epidermis, Fig. 3(1))	**Nitida group**...**10**
–	Old leaf sheaths lacerated (leaves never with a multilayered collenchyma below the abaxial epidermis, or when present then interrupted by a sclerenchyma girder connecting the vascular bundle to the epidermis)	**Bifida group...14**
10	Plants caespitose, at least 0.5 m tall (leaf anatomy with adaxial ribs, and the adaxial surface papillate)	**11**
–	Plants usually forming vegetable hedgehogs (spiny cushions), rarely caespitose, up to 0.5 m tall	**12**
11	Tussocks up to 2.3 m tall; old sheaths remaining intact; inflorescence branches nitid to scaberulous, nodes villous; lemmas villous overall with callus indumentum longer than lemma hairs	***Cortaderia nitida***
–	Tussocks up to 1.5 m tall; old sheaths shattering transversely; inflorescence branches and nodes scabrid; lemma indumentum sometimes only basal with callus indumentum only as long as the lemma hairs	**9. *Cortaderia boliviensis***
12	Leaves densely pilose (leaves folded double, no adaxial ribs)	**10. *Cortaderia sericantha***
–	Leaves glabrous (leaves expanded, with adaxial ribs)	**13**
13	Plants caespitose; sheaths remaining intact; inflorescence branches villous; glumes less than 15 mm long; lemma setae, excluding lobes, to 1.5 mm long; from marshlands in Colombia (anatomy not known)	**11. *Cortaderia pungens***
–	Plants cushion-forming; sheaths splitting transversely; inflorescence branches scabrid; glumes more than 15 mm long; lemma setae, excluding lobes, at least 2 mm long; from epilithic habitats in Peru (leaves without abaxial collenchyma)	**12. *Cortaderia echinata***
14	Leaf upper surface, directly above the ligule, glabrous (leaves abaxially shallow grooves with collenchyma in the grooves (Fig. 3(2))	**15**
–	Leaf upper surface, directly above the ligule, villous (leaves abaxially not grooved, with a weakly developed sclerenchyma layer below the abaxial epidermis)	**16**
15	Leaves not pungent, more than 20 cm long, when dry expanded, disarticulating from the sheath; inflorescences plumose, pedicels not obscured by spikelets; lemma awn 6–17 mm long	**13. *Cortaderia bifida***
–	Leaves pungent, to 20 cm long, when dry folded double, persistent on sheath; inflorescence contracted, pedicels obscured by spikelets; lemma awn 4–8 mm long (anatomy not known)	**14. *Cortaderia planifolia***
16	Glumes 10–22 mm long; lemma setae 1–3 mm long; from Andes	**15. *Cortaderia hapalotricha***
–	Glumes 5–13 mm long; lemma setae 0–2 mm long; from Andes or tepuis	**17**
17	Lemma indumentum 3–4 mm long; setae 3–9 mm; from Andes	**16. *Cortaderia columbiana***
–	Lemma indumentum 4–6 mm long; setae 0–2 mm; from tepuis	**17. *Cortaderia roraimensis***

**Figure 2.**
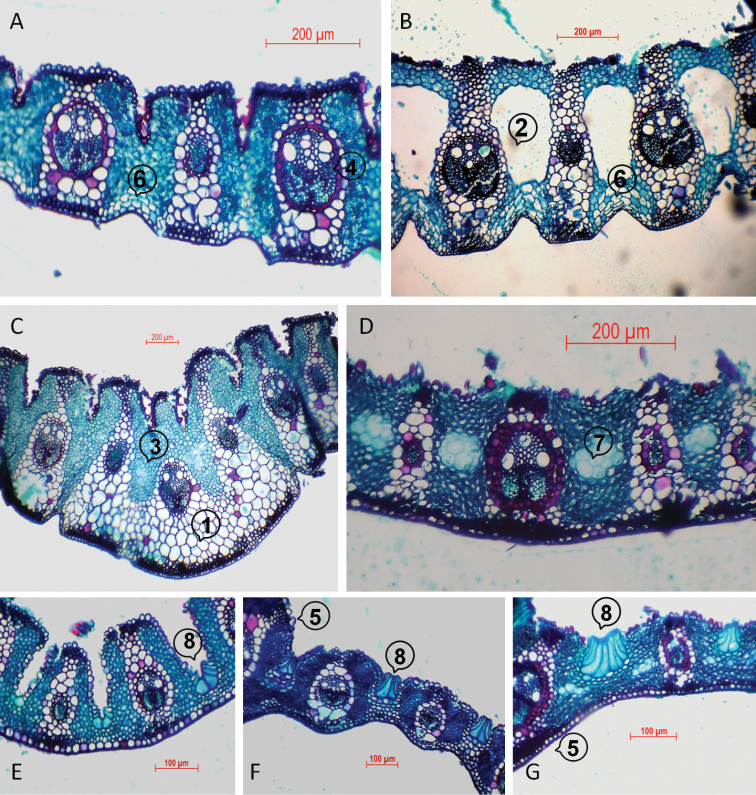
Leaf anatomy of *Cortaderia*, as evident from transverse sections. **A–B** *Cortaderia selloana* (Villamil 11738) **C** *Cortaderia speciosa* (Testoni 644) **D** *Cortaderia vaginata* (Zanín 1654). Comparison of bulliform cells in Egmontiana group: **E** *Cortaderia egmontiana* (Testoni 634) **F** *Cortaderia modesta* (Carauta 927) **G** *Cortaderia vaginata* (Zanín 1654). Structures referred to in the descriptions are labelled as follows: 1, multi-layered abaxial sub-epidermal collenchyma layer; 2, aerenchyma; 3, chlorenchyma; 4, primary vascular bundle; 5, midrib; 6, colourless cells; 7, empty cells; 8, bulliform cells.

**Figure 3.**
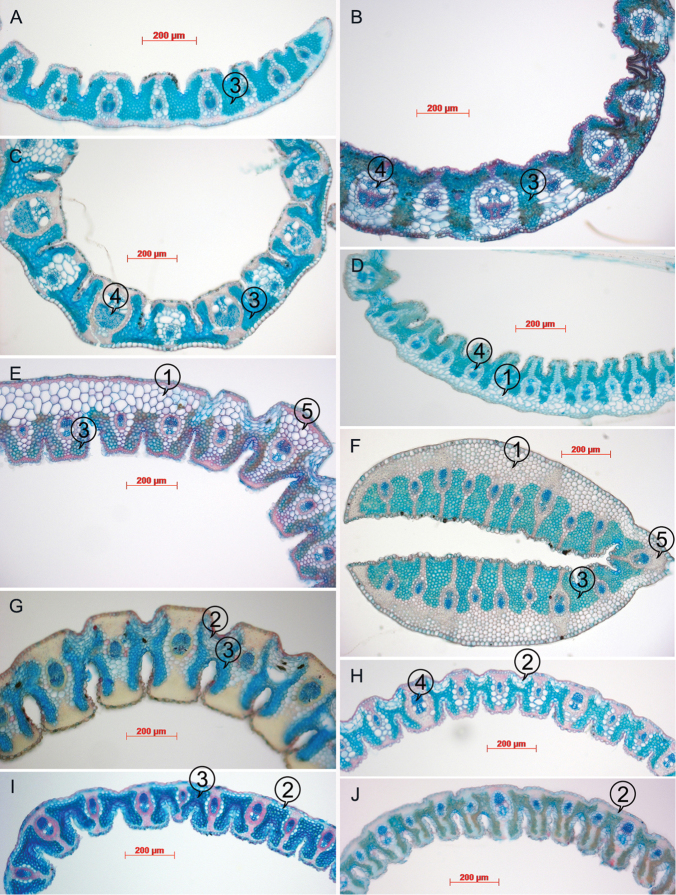
Leaf anatomy of *Cortaderia*, as evident from transverse sections. **A** *Cortaderia egmontiana* (Moore 2677) **B** *Cortaderia modesta* (Glaziou 17913) **C** *Cortaderia hieronymi* (Garcia 563) **D** *Cortaderia nitida* (Laegaard 53121) **E** *Cortaderia boliviensis* (Beck 11273); **F** *Cortaderia sericantha* (Ramsay 967); **G** *Cortaderia echinata* (Peterson 21587) **H** *Cortaderia bifida* (Renvoize 4202) **I** *Cortaderia hapalotricha* (Laegaard 53305) **J** *Cortaderia roraimensis* (Maguire 60448). Structures referred to in the descriptions are labelled as follows: 1, multi-layered abaxial sub-epidermal collenchyma layer; 2, adaxial islands of collenchyma in the abaxial grooves; 3, chlorenchyma; 4, primary vascular bundle; 5, midrib.

#### Notes on species

##### Selloana group

In this group three morphologically and anatomically similar species, with both gynodioecious and apomictic breeding systems, are included. They are easily distinguishable from other species in the genus: they form big tussocks 1.5 to 3 m in diameter and up to 4 m in height, and the leaf edges are strongly cutting. The panicles are large and plumose, very showy, and much larger than in most of the other species. The spikelets have 1-veined glumes, the lemmas are long-acuminate, with or without evident awns, unlobed, 3-veined and with long hairs only in female plants (hermaphrodites glabrous).

The leaf, in transversal section (Fig. 2A–C), is ribbed, with moderately deep square ribs on adaxial and ribbed on abaxial surface. Colourless chlorenchya cells occur between the vascular bundles on the abaxial epidermis. Aerenchyma is sometimes present (Fig. 2B) in all species.

###### 1. Cortaderia selloana

Taxon classificationPlantaePoalesPoaceae

(Schult. & Schult. f.) Asch. & Graebn., Syn. Mitteleur. Fl. 2(1): 325. 1900.

Arundo dioeca Spreng., Syst. Veg. (ed. 16) 1: 361. 1825 [1824], nom. illeg. (non Lour. 1790); Cortaderia dioeca (Spreng.) Speg., Anales Mus. Nac. Buenos Aires 7: 194. 1902; Arundo selloana Schult. & Schult. f., Mant. 3(1): 605. 1827; Gynerium argenteum Nees, Agrost. Bras. 462. 1829, nom. illeg.; Moorea argentea (Nees) Lemaire, Ill. Hort. 2: 14. 1855; Cortaderia argentea (Nees) Stapf, Gard. Chron. ser. 3, 22: 396. 1897, nom. illeg. Type. Uruguay. Montevideo, I-1836, *F. Sellow 570* (lectotype, designated here: B 10 0185657! (http://ww2.bgbm.org/Herbarium/specimen.cfm?Barcode=B100185657); isolectotypes: BAA frag. ex B!, FR photo!).

####### Etymology.

selloana: In honour of Friedrich Sellow (1789–1831), German botanist, a major collector of Brazilian flora.

####### Nomenclatural comments.

Arundo dioeca Spreng. is a later homonym of Arundo dioica Lour. (1790) from Indochina, and is consequently illegitimate. Arundo selloana Schult. & Schult.f. is a new name for Arundo dioica Spreng.; Arundo dioeca Spreng. is cited in the protologue, and the diagnoses are identical. Furthermore, both description cite a Sellow collection, without number, from Montevideo. It is most likely that the type is *Sellow 570* from Montevideo, which is in B, and is designated here as lectotype. Curiously, Conert (1961) proposed *Sellow 396* from Brasilia as holotype of Arundo dioica, although Sprengel explicitly mentions that the type is from Monte Video. Gynerium argenteum Nees is also based on the same collection as Arundo dioica Spreng., plus some additional material. All three names are based on the same type.

###### 1a. Cortaderia selloana subsp. selloana

Taxon classificationPlantaePoalesPoaceae

Figs 1A , 2A–B

####### Common names.

pampas grass, cortadera, cola de zorro, carrizo de las pampas. The origins of the popular name “pampas grass” are somewhat obscure, and do not reflect the ecology of the species (Stapf 1897).

####### Taxonomy.

Cortaderia selloana ssp. selloana can be diagnosed by the glumes about as tall as the basal lemma, and lemma without a distinct awn. The plants are generally larger than those of *Cortaderia araucana* and *Cortaderia speciosa*, and the panicles are larger (0.5 to 1 m long), more lax, and coloured white, pink or yellowish. The similar size of basal lemmas and glumes (6–15 mm) further separates it from *Cortaderia araucana* (glumes 9–17 mm long, ca. ½ length of basal lemmas), whereas the larger glumes separate it from *Cortaderia speciosa* (glumes 6–8 mm, ca. ¾ length of basal lemmas). The large size may also lead to confusion with *Cortaderia nitida*, but it is easily separated by the larger and laxer panicles, 3-veined, awnless lemmas that are glabrous on hermaphrodite plants; and female plants with tiny staminodes. For the distinction from ssp. jubata see below.

Cortaderia selloana ssp. selloana was originally described as dioecious, but Astegiano et al. (1995) showed that it is gynodioecious, and Testoni and Villamil (2014) recorded several populations with only pistillate individuals (so presumably apomictic) in central and northern Argentina. This subspecies presents the greatest morphological variability and geographical range in the genus. The morphological characterization is also complicated by interbreeding between natural populations and cultivated plants.

###### 1b. Cortaderia selloana subsp. jubata

Taxon classificationPlantaePoalesPoaceae

(Lemoine) Testoni & Villamil, Darwiniana, nueva serie 2(2): 272. 2014.

Gynerium jubatum Lemoine, Rev. Hort. 50: 449. 1878; Cortaderia jubata (Lemoine) Stapf, Bot. Mag. 124: t. 7607. 1898. Type: Ecuador, “sent by Lemoine of Nancy and collected at Chimborazo by the botanical collector Roezl”, sine data, B. Roezl s.n. (lectotype designated by Connor & Edgar, Taxon 23: 598 (1974): K 000307978!).

####### Etymology.

*jubata* (Lat.): Having mane, crest, in allusion to the panicle.

####### Common names.

pink pampas grass, jubata grass, cortadera

####### Taxonomy.

This subspecies is generally similar to ssp. selloana, and includes all the morphologically homogenous apomictic populations of the Yungas region. It can be separated from ssp. selloana by the inflorescences which extend far beyond the foliage, and the pink, 75–90 cm long, very lax, pyramidal and nodding panicles. In Ecuador it is sympatric with *Cortaderia nitida*, from which it can be separated by its larger size and its spectacular pink panicles. They can also easily be distinguished by the leaves: in subsp. jubata they are flat and folded V-shaped, while in *Cortaderia nitida* leaves are inrolled from both margins.

###### 2. Cortaderia araucana

Taxon classificationPlantaePoalesPoaceae

Stapf, Gard. Chron. ser. 3, 22: 396. 1897.

Fig. 1B

Moorea araucana (Stapf) Stapf, Gard. Chron. ser. 3, 34: 400. 1903. Type: Chile, llanos de Valdivia, 20-XII-1852, W. Lechler 613 (lectotype designated by Connor & Edgar, Taxon 23: 598 (1974): K 000308157!; isolectotypes: P photo!, W photo!, US! fragm. ex K).
Cortaderia quila var. patagonica Speg., Anales Mus. Nac. Buenos Aires 7: 194. 1902. Type: Argentina, Chubut, “non rara in rupestribus secus Carren-leofú, aest. 1899-900”, N. Illín s.n. (lectotype, here designated: LP!).
Cortaderia longicauda Hack., Repert. Spec. Nov. Regni Veg. 10 (243–247): 169. 1911. Type: Chile, Valdivia, “Potrero Coihue, I-1861”, R. A. Philippi s.n. (lectotype designated as holotype by Connor & Edgar, Taxon 23: 598 (1974): W-1916-0039626 (http://jacq.nhm-wien.ac.at/djatoka/jacq-viewer/viewer.html?rft_id=w_19160039626&identifiers=w_19160039626); isolectotype: BAA!).
Cortaderia araucana var. fuenzalidae Acevedo, Bol. Mus. Nac. Hist. Nat. Santiago de Chile 27(4): 239. 1959. Type: Chile, Curico, Potrero Grande, 5-XI-1943, M. Espinosa s.n. (lectotype, here designated: SGO photo!).
Cortaderia araucana var. skottsbergii Acevedo, Bol. Mus. Nac. Hist. Nat. Santiago de Chile 27(4): 240. 1959. Type: Chile, provincia Chiloé, región del Corcovado, sine data, C. Reiche s.n. (lectotype, here designated: SGO photo!).

####### Etymology.

-*ana*, indicating connection. From the Araucania region of Chile.

####### Common names.

cortadera

####### Taxonomy.

In the Selloana group, *Cortaderia araucana* is readily diagnosed by the basal lemmas longer than 12.5 mm and much longer than the glumes. The spikelets are 20–35 mm long and the lemma of the basal floret 14–25 (30) mm long (including awn of 5–11 mm long). The species is found in the southern (austral) Andean region.

*Cortaderia araucana* includes extensive morphological variation, and both gynodioecious and apomictic populations. This variability led Acevedo Vargas (1959) to recognize three varieties, which are no longer maintained. In northern Patagonia *Cortaderia araucana* and *Cortaderia selloana* are sympatric, but the plants of *Cortaderia araucana* are somewhat smaller, with less lax panicles and flowering in the austral spring (late November and early December), whereas *Cortaderia selloana* flowers in the austral summer (January and February). Further, the spikelets are different: the glumes are shorter than the basal floret, the lemma may terminate in an awn that arises between two lower lateral setae. The leaf anatomy of both species is similar.

###### 3. Cortaderia speciosa

Taxon classificationPlantaePoalesPoaceae

(Nees & Meyen) Stapf, Gard. Chron. Ser. 3(22): 396. 1897.

Fig. 1C

Gynerium speciosum Nees & Meyen, Nov. Act. Nat. Cur. 19 suppl. 1: 153. 1843; Gynerium argenteum var. strictum E. Desv., Fl. Chile. 6: 328. 1854; Moorea speciosa (Nees & Meyen) Stapf, Gard. Chron. Ser. 3, 34: 400. 1903. Type: Chile, ad flumen Copiapo dictum circa Nantoco in provincia Copiapó reipublicae Chilensis, III-1831, F. J. F. Meyen s.n. (lectotype designated by Connor & Edgar, Taxon 23: 603 (1974): B 10 0217503! (http://ww2.bgbm.org/Herbarium/specimen.cfm?Barcode=B100217503); isolectotype: K! frag. ex B).
Gynerium quila Nees & Meyen, Nov. Act. Nat. Cur.19 suppl. 1: 153. 1843; Cortaderia quila (Nees & Meyen) Stapf, Gard. Chron. Ser. 3: 22: 396. 1897; Moorea quila (Nees & Meyen) Stapf, Gard. Chron. Ser 3, 34: 400. 1903. Type: Chile, ad Copiapó fluvium circa Nantoco, sine data, F. J. F. Meyen s.n. (syntype: B!); Perú, ad lacum Titicacam et ad pedem vulcani Arequipensis. Femina planta. Mascula ignota est., 1000 m, Maio, F. J. F. Meyen s.n. (syntype: B 10 0217504 (http://ww2.bgbm.org/Herbarium/specimen.cfm?Barcode=B100217504; isosyntype: BAA! frag. ex B).
Gynerium quila var. pygmaeum Meyen, Nov. Act. Nat. Cur. 19 Suppl. 1: 153. 1843. Type: Perú, “ad lacum Titicacam. ♀”, IV-1841, F. J. F. Meyen s.n. (lectotype, designated here: B 10 0217506! (http://ww2.bgbm.org/Herbarium/specimen.cfm?Barcode=B100217506).
Gynerium argenteum var. parviflorum E. Desv., Fl. Chile. 6: 328. 1854. Type: Chile, Mal Paso, cordillera de Guanta, a la orilla de los arroyos, 2490 m., en donde forma copas apretadas de un metro y más, sine data, C. Gay s.n. (lectotype, designated here: P 00506920!).
Gynerium atacamense Phil., Linnaea 33: 289.1865. Cortaderia atacamensis (Phil.) Pilg., Bot. Jahrb. 37: 374. 1906. Type: Chile, prope San Pedro de Atacama, I-1854, R. A. Philippi s.n. (lectotype, designated as holotype by Connor & Edgar, Taxon 23: 597 (1974): SGO photo!; isolectotype: BAA! frag. ex SGO, W!).
Cortaderia rudiuscula Stapf, Gard. Chron. Ser. 3, 22: 396. 1897. Moorea rudiuscula (Stapf) Stapf, Gard. Chron. Ser. 3, 34: 400. 1903. Type: Chile, Santa Rosa de los Andes, V-1882, J. Ball s.n. (lectotype, designated by Connor & Edgar, Taxon 23: 601 (1974): K!; isolectotype: BAA! frag. ex K).

####### Etymology.

*speciosus* (Latin), beautiful, showy.

####### Nomenclatural comments.

The binomials *Gynerium speciosum*, *Gynerium neesii* and *Gynerium pygmaeum* – mentioned as new species by Meyen (1834), from Copiapo (Chile) and Lake Titicaca (Peru), respectively – are synonyms of *Cortaderia speciosa*, but are invalid (nomina nuda) as no descriptions were published. Their identity can be determined, because the specimens in B! were annotated with the Meyen names. *Gynerium speciosum* was validated by Nees in 1943. Tropicos (Downloaded 14 December 2016) lists the species as described by Nees in 1841 (Nees ab Esenbeck 1841), but this is erroneous. Conert (1961) designated *Philippi 1024* (B photo!) from Chile (“Atacama oppidum, 1824”) as lectotype of *Gynerium atacamense* Phil. However, the type has been found in the herbarium SGO (Connor, 1983) and, therefore, the lectotype designated by Conert should not be taken into account. The binomial *Arundo quila* Molina is a synonym of *Chusquea quila* Kunth (Bambusoideae). In some works, it has been confused *Gynerium quila* Nees & Meyen (basionym of *Cortaderia quila* Nees & Meyen) Stapf, therefore, the binomials *Gynerium quila* (Molina) Nees & Meyen and *Cortaderia quila* (Molina) Stapf are invalid.

####### Common names.

cortadera

####### Taxonomy.

In the Selloana group, *Cortaderia speciosa* can be diagnosed by the short basal lemmas, which are less than 13 mm long. The spikelets are 8–15 mm long and the basal lemma 7.0–12.5 mm long (including awn, 1–4 mm). It differs from other species in the group by its very compact, bright brown panicles with ascending, short and stiff branches. The species is readily distinguished by the small floret sizes. The leaf anatomy is also somewhat different from the other species of the group (Fig. 2C): the midrib is rounded and somewhat lower; the outer sheath of the central vascular bundle without projections to the adaxial epidermis; and with a massive abaxial sub-epidermal collenchyma layer, only in the middle part of the leaf. The latter occurs in the Nitida group but along the leaf. It is known only by pistillate plants from desert regions (the Puna) of Argentina, Bolivia and Chile.

This species is completely apomictic, and several morphological subgroups can be recognized. As these are all apomicts, it is presumed that they derive from the same ancestral sexual population. The material previously separated as *Cortaderia rudiuscula* has longer (9–12 mm) and more slender lemmas, than the material previously separated as *Cortaderia speciosa* (lemmas ca. 8 mm), but there is no clear separation between these two forms.

##### Lamprothyrsus group

This group is very distinct within *Cortaderia*. Morphologically, it differs by the long, filiform awns, 14–35 mm long; glumes without veins; and by the sheaths which are always intact. Furthermore, the leaf anatomy differs by the primary vascular bundles with lignified sheaths and girders, tertiary vascular bundle sheaths and girders collenchyma (Fig. 3C). The group includes only one species. The enormous variation with this species complex could be due to its apomictic reproduction (Connor and Dawson 1993).

###### 4. Cortaderia hieronymi

Taxon classificationPlantaePoalesPoaceae

(Kuntze) N.P.Barker & H.P.Linder, Ann. Missouri Bot. Gard. 97(3): 342. 2010.

Figs 1G , 3C

Triraphis hieronymi Kuntze, Revis. Gen. Pl. 3(3): 373. 1898; Danthonia hieronymi (Kuntze) Hack., Anales Mus. Nac. Buenos Aires ser. 3, 6: 484. 1906; Lamprothyrsus hieronymi (Kuntze) Pilg., Bot. Jahrb. Syst. 37 (Beibl. 85): 58. 1906. Type: Argentina, Córdoba, “prope urbem”, 6 Nov. 1881, G. H. E. W. Hieronymus s.n. (lectotype, designated as holotype by Conert, Syst. Anat. Arundineae 128 (1961): B!; isolectotype: K!).
Triraphis hieronymi var. jujuyensis Kuntze, Revis. Gen. Pl. 3(3): 374, 1898; Danthonia hieronymi var. jujuyensis Kuntze, Anales Mus. Nac. Buenos Aires ser. 3, 6: 486 (1906); Lamprothyrsus hieronymi var. jujuyensis (Kuntze) Pilg., Bot. Jahrb. Syst. 37 (Beibl. 85): 59, 1906. Type: Argentina, Jujuy, sine data, O. Kuntze s.n. (lectotype, designated as holotype by Conert, Syst. Anat. Arundineae 130 (1961): B!).
Lamprothyrsus hieronymi var. pyramidatus Pilg., Bot. Jahrb. Syst. 37 (Beibl. 85): 59. 1906. Type: Bolivia, ad Toldos prope oppium Bermejo, 2000m, 8 Dec. 1903, K. A. G. Fiebrig 2372 (lectotype, designated as holotype by Conert, Syst. Anat. Arundineae 128 (1961): B 10 0249138! (http://ww2.bgbm.org/Herbarium/specimen.cfm?Barcode=B100249138); isolectotypes: K, US).
Lamprothyrsus hieronymi var. nervosus Pilg., Bot. Jahrb. Syst. 37 (Beibl. 85: 59. 1906. Type: Argentina, Cordoba, Sierra Achala, 11 Nov. 1878, G. H. E. W. Hieronymus 43 (lectotype, designated as holotype by Conert, Syst. Anat. Arundineae 129 (1961): B 01 0272938! (http://ww2.bgbm.org/Herbarium/specimen.cfm?Barcode=B100272938); isolectotype: W).
Lamprothyrsus hieronymi var. tinctus Pilg., Bot. Jahrb. Syst. 37 Beibl. 85: 59. 1906. Type: Bolivia, Bermejo, 1400m, 16 Nov. 1903, K. Fiebrig 2099 (lectotype, designated as holotype by Conert, Syst. Anat. Arundineae 129 (1961): B 10 0249137! (http://ww2.bgbm.org/Herbarium/specimen.cfm?Barcode=B100249137); isolectotypes: K, L!, US!).
Lamprothyrsus peruvianus Hitchc., Proc. Biol. Soc. Washington 36: 195. 1923; Cortaderia peruviana (Hitchc.) N.P.Barker & H.P.Linder, Ann. Missouri Bot. Gard. 97(3): 342. 2010. Type: Peru, Yanahuanca, 16–22 Jun 1922, J. F. Macbride & W. Featherstone 1205 (lectotype, designated as holotype in F: F-V0040645F, photo F-50163 (http://emuweb.fieldmuseum.org/web/pages/common/imagedisplay.php?irn=39615&reftable=efmnh&refirn=257048); isolectotypes: US photo!, K!).
Lamprothyrsus venturi Conert, Syst. Anat. Arundineae 130. 1961. Type: Argentina, prov. Tucuman, Famailla, Villa Nougues, 21-10-1923., S. Venturi 2534 (lectotype, designated as holotype by Conert, Syst. Anat. Arundineae 131 (1961): K; isotype: US!).

####### Etymology.

In honour of George Hans Emmo Wolfgang Hieronymus (1846–1921), German botanist, sometimes resident of Argentina.

####### Common names.

Seringuilla, sivinga (Tucuman).

####### Taxonomy.

This species contains substantial variation in the robustness of the plants. Conert (1961) partitioned this variation into three species (*Lamprothyrsus peruvianus*, *Lamprothyrsus venturi* and *Lamprothyrsus hieronymi*) and Pilger (1906) recognized varieties in his *Lamprothyrsus hieronymi*. Study of the herbarium material suggests that this is most likely all one taxon (Bernardello 1979), but an analysis of variation within natural populations in the field would be useful to understand the range of variation possible. *Cortaderia hieronymi* differs from the other species in *Cortaderia* by the very long hair-like lemma awns and setae, the glumes without veins, and the small flowers with relatively short and sparse lemma hair.

Only apomictic populations are known, but a few fertile staminate specimens with long hairs on the lemmas were found (Bernardello 1979). It is not known if they can form viable caryopses, and if the species is dioecious or gynodioecious.

In the central and northern Argentina to Ecuador *Cortaderia hieronymi* is sympatric with the two subspecies of *Cortaderia selloana*, but it is easily separated by its smaller panicles, spikelets with glumes without veins, and 5-veined, 3-awned lemmas. In Peru and Ecuador it is sympatric with *Cortaderia bifida*, with which it is often confused: in both species the old leaf sheaths are lacerated and the spikelets have long awns, but the spikelets of *Cortaderia hieronymi* are bigger, and the lemmas with longer and robust central awns.

##### Egmontiana group

This group includes three quite distinctive species. *Cortaderia vaginata* and *Cortaderia modesta* have an unusual (for *Cortaderia*) leaf anatomy lacking ribs, and with deeply split phloem poles (Fig. 3B), and large bulliform cells (Fig. 2E–G), which are rare in the other groups.

###### 5. Cortaderia egmontiana

Taxon classificationPlantaePoalesPoaceae

(Roem. & Schult.) M.Lyle ex Connor, Darwiniana 49: 90. 2011.

Figs 1E , 2E , 3A

Arundo egmontiana Roem. & Schult., Syst. Veg., ed. 15 b [Roemer & Schultes] 2: 511. 1817. Phragmites egmontiana (Roem. & Schult.) Trin. ex Steud., Nomen. Bot. (ed. 2) 2: 324. 1840. Type: Falkland / Malvinas Islands, Port Egmont, R. J. Schuttleworth s.n. (type: BM photo!).
Arundo pilosa d’Urv., Mém. Soc. Linn. Paris 4: 603. 1826; Cortaderia pilosa (d’Urv.) Hack. ex Dusén, Bol. Acad. Nac. Ci. 16: 253. 1900; Gynerium pilosum (d’Urv.) Macloskie in Scott, Rep. Princeton Univ. Exped. Patagonia, Botany 8, part 1: 213. 1904; Phragmites pilosa (d’Urv.) Macloskie & Dusén in Scott, Rep. Princeton Univ. Exped. Patagonia, Botany 8, suppl. bot.: 50. 1915. Ampelodesmos australis Brongn. in Duperrey, Voy. Monde 2(2): 31. 1829, nom. illeg. Type: Falkland / Malvinas Islands, 1825, J. S. C. D. D’Urville s.n. (central inflorescence designated as lectotype by Connor & Edgar, Taxon 23: 600 (1974): P 00740221! (http://mediaphoto.mnhn.fr/media/1443644100310dGB3ZqFqm8JGPDaz; isolectotype: B!).
Calamagrostis patula Steud., Syn. Pl. Glumac. 1(6): 422. 1854. Type: Chile, Huiti, sine data, W. Lechler 760 (lectotype, selected here: P-00740220 (http://mediaphoto.mnhn.fr/media/1443644088798jsLY8AS29Euj4oHx); isolectotypes: GOET; W photo!)
Poa phragmites Phil., Anales Univ. Chile 43: 576. 1873. Type: Chile, volcan de Osorno, 1872, C. Juliet s.n. (holotype: SGO photo! (http://plants.jstor.org/stable/viewer/10.5555/al.ap.specimen.sgo000000667; isotype: BAA! frag. ex SGO);
Gynerium nanum Phil., Anales Univ. Chile 94: 155. 1896. Type: Falkland / Malvinas Islands, Dec. 1884, C. Martin s.n. (lectotype, designated as holotype by Connor & Edgar, Taxon 23: 600 (1974): SGO 065328!; isolectotype: BAA!).
Calamagrostis scirpiformis Phil., Anales Univ. Chile 94: 20. 1896. Type: Chile, ad lacum Llanquihue, I-1866, F. Philippi s.n. (lectotype, designated here: SGO 37097; isolectotypes: US, BAA!)
Cortaderia minima Conert, Syst. Anat. Arundineae 119. 1961; Cortaderia pilosa var. minima (Conert) Nicora, Darwiniana 18(1–2): 80. 1973. Type: Chile, Andes, Villarrica, “in feuchten Schluchten nahe der Waldgrenze”, 1897, F. W. Neger s.n. (lectotype, designated as holotype by Conert, Syst. Anat. Arundineae 119 (1961): M; isolectotypes: W5945! B! fragm. ex M).

####### Etymology.

*egmontiana*: called after Port Egmont in the Falklands / Malvinas Islands.

####### Nomenclatural comments.

Brongniart (1829) described *Ampelodesmos australis*, and explicitly included *Arundo pilosa* D’Urville as a synonym, noting that this species is better placed in *Ampelodesmos*.

####### Taxonomy.

The species can be readily diagnosed by the combination of compact inflorescences, almost glabrous leaves, and either no, or poorly developed, awns and setae on the lemmas. The habit and dense inflorescences are as in *Cortaderia sericantha*, but *Cortaderia egmontiana* differs by the absence of setae, and by the almost completely glabrous leaves. The lemma and spikelet morphology (reduced or absent awns and setae) suggests an affinity to the eastern Brazilian species *Cortaderia vaginata* and *Cortaderia modesta*. From these two species *Cortaderia egmontiana* can be separated by the compact inflorescences and the tendency of the leaf blades to disarticulate from the sheaths. It is the only *Cortaderia* species in southern South American temperate zone. The leaf anatomy (Figs 2E, 3A) does not show any distinctive peculiarities.

There is remarkable intraspecific variation in the spikelet and floret sizes, and Conert (1961) separated the forms with smaller spikelets as *Cortaderia minima*. Moore (1983) suggested that the two taxa were latitudinally separated, with the southern populations constituting *Cortaderia pilosa*, and the northern *Cortaderia minima*. On the available material, there is indeed a break in the glume length variation. However, this fits no ecological or geographical pattern, and both small and large-glume forms occur in both the Falkland / Malvinas islands and Tierra del Fuego. Further north, indeed, only the small-glume form is found. This suggests that this size variation has no biological significance, accordingly it is ignored here.

###### 6. Cortaderia modesta

Taxon classificationPlantaePoalesPoaceae

(Döll) Hack., Ark. Bot. 9(5): 4. 1909.

Figs 1F , 2F , 3B

Gynerium modestum Döll, Fl. Bras. [Martius] 2(3): 240. 1880. Type: Brasil, near Rio de Janeiro, Serra dos Órgãos, au Frade (2 ou 3 mois après l’incendie de la forêt), 11-X-1869, A. F. M Glaziou 4352 (lectotype, designated by Connor & Edgar, Taxon 23: 600 (1974): W 10406!; isolectotypes K!, NY!).
Gynerium ramosum Hack., Arq. Mus. Nac. Rio de Janeiro 13: 73. 1903. Gynerium modestum f. ramosa (Hack.) Hack., Ark. Bot. 9(5): 4. 1909. Type: Brasil, Campo 2100 m, 18 Dec. 1895, P. K. H. Dusén s.n. (lectotype, designated here: W!).

####### Etymology.

*modesta* (Latin) = moderate, presumably referring to the culms of average height.

####### Nomenclatural comments.

The locality information given by Connor and Edgar (1974) is incorrect. Note that Glaziou made several collections of the same species from the same area.

####### Common names.

cabeça de negro, capim-de-anta.

####### Taxonomy.

Some specimens show a poorly developed axillary inflorescence developed at the penultimate node of the flowering culm. The almost awnless lemmas, with the paleas as long as the lemmas, and the very dense callus hairs compared to the short lemma back hairs, are almost unique in the genus. Its closest relative might be *Cortaderia vaginata* from Santa Catarina, further south along the Brazilian Atlantic coast. It is readily distinguished from *Cortaderia vaginata* by the persistent leaf sheaths and the awnless lemmas. According to herbarium labels the plant forms massive tussocks with persistent red, burnt sheaths.

###### 7. Cortaderia vaginata

Taxon classificationPlantaePoalesPoaceae

Swallen, Sellowia 7: 9. 1956.

Figs 1D , 2D,G

####### Type.

Brasil, Santa Catarina, Bom Retiro, Campo dos Padres, 16 Dec. 1948, R. Reitz 2398 (lectotype, designated as holotype by Connor & Edgar, Taxon 23: 603 (1974): US 00133444!; isolectotype: HBR).

####### Etymology.

*vagina* (Latin) = sheath. Possibly referring to the conspicuous leaf-sheaths, a feature that is common to most of the genus.

####### Common names.

Penacho, Capim-Penacho.

####### Taxonomy.

According to Swallen (1956) this species resembles *Cortaderia parviflora*, but differs by the glabrous lemmas and long-villous calli. It is unusual among the species assigned to *Cortaderia* by the glabrous lemma, and the almost glabrous pedicels and inflorescence axes. This species may be a local endemic, and might be quite rare. It is probably most closely related to *Cortaderia modesta*, which also has a reduced awn, but differs by the sheaths which are lacerated, lax panicles (without axillary panicles), and the glabrous lemma. Geographically it can be immediately identified as the only *Cortaderia* species from Santa Catarina in southern Brazil.

The leaf anatomy is identical to that of *Cortaderia modesta*, except that all sections appear to have large empty cells in the middle of the leaf (Fig. 2D), between the vascular bundles. These were seen on some sections of *Cortaderia modesta*, but rarely.

##### Nitida group

The Nitida group can be characterized by the leaf sheaths which generally remain intact, and the leaves which, in transverse section, show a massive abaxial sub-epidermal collenchyma layer. *Cortaderia nitida*, *Cortaderia pungens* and *Cortaderia boliviensis* are very similar, whereas *Cortaderia sericantha* is quite distinct by the villous, folded leaves with no adaxial ribs. The distinction of *Cortaderia pungens* is not clear, and needs fieldwork. The new *Cortaderia echinata* is also included in here although anatomically it fits into the next group. Leaf anatomically, *Cortaderia nitida* and *Cortaderia boliviensis* are very similar, with papillate adaxial surfaces, deep adaxial grooves, and a well developed abaxial collenchyma layer. The leaf anatomy of *Cortaderia pungens* is not known.

###### 8. Cortaderia nitida

Taxon classificationPlantaePoalesPoaceae

(Kunth) Pilg., Bot. Jahrb. Syst. 37: 374. 1906.

Figs 1H , 3D

Arundo nitida Kunth in Humb. et Bonpl., Nov. Gen. Sp. [H.B.K.] 1: 149. 1816; Gynerium nitidum (Kunth) Pilg., Bot. Jahrb. Syst. 27: 31. 1899. Type: Colombia, inter Guachucal et Tuqueres, sine data, A. J. A. Bonpland s.n. (lectotype, designated as holotype by Connor & Edgar, Taxon 23: 600 (1974): B; isolectotypes: BM, K!).
Cortaderia sodiroana Hack., Oesterr. Bot. Z. 52: 238. 1902. Type: Ecuador, in reg. silvat. suband., 1872, L. Sodiro s.n. (lectotype, designated by Connor & Edgar, Taxon 23: 600 (1974): W 25246!; isolectotype: US!). A second Sodiro collection, same date and place, on the same sheet as the lectotype [W 25245], does not belong to Cortaderia nitida, and does not fit Haeckel’s description.

####### Etymology.

*niteo* (Latin) = shine. It may refer to the persistently intact, more or less white, leaf sheaths.

####### Common names.

“Sigse de Páramo”.

####### Taxonomy.

*Cortaderia nitida* is a distinctive grass. It is the tallest and most robust species of this group. The lamina margins are inrolled. The basal sheaths gradually become shorter with age, but do not become lacerated, the leaf blades are scabrid in the upper half but not the lower, and the inflorescence branches which are scaberulous while the pulvini often have a few long hairs (the latter seems to be unique in the genus). The callus usually has very long spreading hairs (more than 2 mm, almost equivalent to the lemma hairs), and the setae are less than 2 mm long. The other tall *Cortaderia*, *Cortaderia bifida*, has central awns that are longer than 8 mm, and very well developed setae. The lemma shape is similar to *Cortaderia columbiana*, but the inflorescence branches are scaberulous in *Cortaderia nitida*, and villous in *Cortaderia columbiana*.

This species also approaches the Selloana group by it large size, big plumose inflorescences, and especially by the lemma shape. It is easy to confuse the lemmas of the two groups, but in Nitida group the lemmas are 5–7 veined, hairy in both sexes, while in Selloana group the lemmas are 3-veined, hairy in female plants and glabrous in hermaphrodite plants. The plastid sequence data also places this species as sister to the Selloana group, but this is not corroborated by the ITS-based phylogeny.

Laegaard (1997) mentions a distinct form of smaller and more delicate plants from the province of Azuay in Ecuador, and with three-nerved glumes, but we have not seen any material of it.

The leaf anatomy (Fig. 3D) is similar to that of *Cortaderia boliviensis*, and approaches that of *Cortaderia sericantha*. A well-developed layer of collenchyma is found below the abaxial epidermis, and overall there is little evidence of lignification. It differs from *Cortaderia sericantha* by the well-developed adaxial grooves and the not quite so massive collenchyma, and by the presence of adaxial papillae.

###### 9. Cortaderia boliviensis

Taxon classificationPlantaePoalesPoaceae

M.Lyle, Novon 6(1): 72. 1996.

Fig. 3C

Cortaderia bifida Pilg. var. grandiflora Henrard, Meded. Rijks-Herb. 40: 67. 1921. Type: Bolivia, Departamento Cochabamba: “Charactergrass der Andenwiesen über Tablas, feuchte Stellen, 3400 m, Mai 1911, T. C. J. Herzog 2194 (holotype: L; isotypes: S, US!, W!, Z!).

####### Etymology.

-*ense* (Latin), denoting origin. From Bolivia.

####### Taxonomy.

This species is very similar to *Cortaderia nitida*, with which it shares the (usually) non-lacerated, entire leaf sheaths and the shape of the lemmas, as well as largely similar leaf anatomy. However, neither chloroplast nor nuclear genome indicates such a relationship for *Cortaderia boliviensis* (Pirie et al. 2009). It differs by the horizontally shattering sheaths. More inconsistent differences are in the indumentum of the floret, with the callus indumentum of *Cortaderia boliviensis* being shorter than in *Cortaderia nitida*. Lyle (1996) diagnosed *Cortaderia boliviensis* against *Cortaderia bifida*, under which it was originally described as a variety by Henrard in 1921. Mostly it is very different from *Cortaderia bifida*: the latter has much longer lemma setae and the basal sheaths are lacerated and not shattered. The type collection, however, is easily confused with *Cortaderia bifida* due to the long awns and setae, and somewhat fragmented leaf sheaths. The leaf anatomy is also quite different.

The leaf anatomy (Fig. 3E) is like that of *Cortaderia nitida*, with adaxial grooves and a well-developed abaxial collenchyma layer. There are differences in detail, and wider sampling may well indicate that this is within-species variation.

###### 10. Cortaderia sericantha

Taxon classificationPlantaePoalesPoaceae

(Steud.) Hitchc., Contr. U.S. Natl. Herb. 24: 348. 1927.

Figs 1I , 3F

Danthonia sericantha Steud., Syn. Pl. Glumac. 1(3): 246. 1854. Type: Ecuador, Quito “On boggy plains on the eastern Cordillera at 13000 feet above sea level”, sine data, W. Jameson 93 (lectotype designated by Connor & Edgar, Taxon 23: 602 (1974): K!; isolectotypes: K! - frag US!, OXF!, TCD!).
Danthonia jubata Sodiro, Revista Colegio Nac. Vicente Rocafuerte 12: 91. 1930. Type: Ecuador, Pinchincha, sine data, A. S. J. Mille s.n. (NY, MO photo!, US!).

####### Etymology.

*serios* (Greek) = silken + *Anthos* (Greek) = flower. Presumably this refers to the silky-haired leaves, a diagnostic trait for this species.

####### Taxonomy.

This species is very distinctive in *Cortaderia* by its very villous leaves, which are rolled rather than flat, and quite pungent; the compact inflorescences with short inflorescence branches; the glumes with three veins and which are much longer than the packet of florets; and the tuft of hair at the base of the spikelets. The inflorescences are similar to those of *Cortaderia egmontiana*, but the villous leaves immediate distinguish our species from *Cortaderia egmontiana*. The intact leaf sheaths, pungent leaf tips, and compact growth form related this species to *Cortaderia pungens* and *Cortaderia echinata*. The remarkably large glumes, much overtopping the packet of florets, are shared with *Cortaderia echinata*.

The leaf anatomy (Fig. 3F) could be unique in the genus. The abaxial half of the leaf, in cross-section, consists of colourless collenchyma. The vascular bundles are very slender, and the girders taper towards the adaxial epidermis. Adaxially the leaves are only very slightly grooved.

###### 11. Cortaderia pungens

Taxon classificationPlantaePoalesPoaceae

Swallen, Contr. U.S. Natl. Herb. 29: 251. 1948.

Danthonia confusa L.B.Sm., Phytologia 22(2): 89. 1971, non Danthonia pungens Cheeseman, 1906. Type: Colombia, Dept. Santander, Páramo de Santurban, near Vetas, 17 Jan. 1927, E. P. Killip & A. C. Smith 17467 (lectotype, designated as holotype by Connor & Edgar, Taxon 23: 600 (1974): US 00133443!; isolectotype: K!).

####### Etymology.

*pungens* (Latin): piercing, terminating in a sharp point. This describes the leaf tips.

####### Taxonomy.

This species is often placed with *Cortaderia hapalotricha*, from which it differs by (a) shorter growth-form (less than 1 m tall); (b) the intact leaf bases; (c) the rolled, pungent leaves; and (d) deeply lobed lemmas. The two species have much in common (leaf anatomy, spikelet and inflorescence structure). It is possible that they are ecotypes of each other, and the problem needs critical field work. We keep them separate on the very different growth-form. The intact leaf bases and pungent leaves suggest a relationship to *Cortaderia sericantha* and *Cortaderia echinata*, but the species is readily separated from these two by the much shorter glumes.

The leaf anatomy was not studied.

###### 12. Cortaderia echinata

Taxon classificationPlantaePoalesPoaceae

H.P.Linder sp. nov.

urn:lsid:ipni.org:names:77159701-1

Figs 3G , 4A–G

####### Type.

Peru, vicinity of Cerro Ayrahnanca pass ca. 1 km E of Lugo Ututo on road between Cataparaco and Utcuyau, 4223 m. Rocky slopes, 11 Mar 2008, *P. M. Peterson, R. J. Soreng, M. I. la Torre & J. V. Rojus Fox 21587* (holotype: Z!, isotype: US!).

####### Diagnosis.

Similar to *Cortaderia pungens* by the small compact habit and pungent leaves, but differing by the shattering leaves and the longer spikelets.

####### Description.

Plants forming tough, perennial cushions (vegetable hedgehogs) to 30 cm in diameter and to 30 cm tall. Basal sheaths white, shiny, persistent, when old splitting transversely into segments, puberulous between the veins. Ligule a dense ring of hairs 2–3 mm long, sheath mouth glabrous. Leaf blades 80–150 × 2–3 mm; C-shaped at base and margins incurved towards apex, forming a rolled, viciously pungent tip; disarticulating from the persistent sheath at the ligule. Inflorescence paniculate, contracted, ovate, 60–100 × 15–25 mm, with 100–300 spikelets; branches and pedicels shorter than and obscured by the spikelets, scaberulous. Female-fertile spikelet 16–22 mm long; with ca. 3 florets. Glumes 16–22 × 0.6–0.8 mm; twice as long as the packet of florets; 1 veined, acute, glabrous, straw to almost white, upper and lower glumes similar. Callus ca. 0.75 mm long; indumentum 2–2.5 mm long, overtopping the base of the lemma hairs length; rhachilla 0.75 mm long. Second lemma ca. 4 mm long, 5 veined, indumentum scattered on lower half of lemma back, about as long as the lemma lobes, 5–6 mm long; lemma-lobes acute, 3–4.5 mm long, setae 2–3 mm long, distinctly shorter than lemma lobes, included in the glumes; awn simple, 8.5–10 mm, longer than setae. Palea linear, 5 × 0.5 mm, obscurely bilobed, keels sinuose; scabrid, with hair-tufts along mid-margins. Lodicules obtriangular and with bristles.

####### Leaf anatomy.

Leaf in transverse section expanded, sclerophyllous; margins gently tapering, sclerenchyma caps well-developed; adaxial furrows located between all vascular bundles, the same over primary and tertiary vascular bundles, about half depth of leaf, forming narrow clefts, ribs flat-topped; abaxial ribs and furrows present. Vascular bundles closer to abaxial surface, 3 primary vascular bundles in half a leaf section, with 1–2 tertiary vascular bundles between the primary vascular bundles. primary vascular bundles elliptical; phloem without lignified cells; metaxylem vessels narrower than outer bundle sheath cells; outer bundle sheath clearly distinct from chlorenchyma, cells larger and colourless, with adaxial and abaxial interruptions; inner bundle sheath walls thickened anticlinally, cells smaller than outer bundle sheath cells; adaxial sclerenchyma as inversely anchor-shaped girders; abaxial sclerenchyma as trapezoidal girders. tertiary vascular bundles outer bundle sheath cells distinct from and larger than chlorenchyma cells, walls thickened anticlinally or all round; with abaxial interruption only; adaxial bundle sheath extension present with cells smaller than outer bundle sheath cells; adaxial sclerenchyma inversely anchor-shaped girders; abaxial sclerenchyma as trapezoidal girders; phloem without lignified cells or with only the inner bundle sheath lgnified. Mesophyll of small, angular isodiametric chlorenchyma cells with small air spaces. Abaxial epidermal cells all larger than adaxial ones; outer wall twice as thick as inner wall; walls equal to mesophyll walls. Subepidermal layer of sclerified fibres only in marginal regions of leaves, absent from the middle of the leaf (directly next to leaf margins), 2-3 cells thick; with large clear parenchymatous cells below abaxial furrow present, connected via collenchyma cells to the adaxial furrow to the epidermis and so partitioning the chlorenchyma. Bulliform cells absent; abaxial epidermal zonation present (Fig. 3G).

####### Etymology.

*echinus* (Latin) = hedge-hog or sea-urchin. The plant is spiny like a hedgehog.

####### Distribution and ecology.

South America, Peru.

####### Altitude.

4220–4230 m.

####### Habitat.

Rock ledges (bedrock slabs); moisture regime: in soil pockets on rock. Forming cushions on almost flat rock slabs, in pockets of soil.

**Figure 4.**
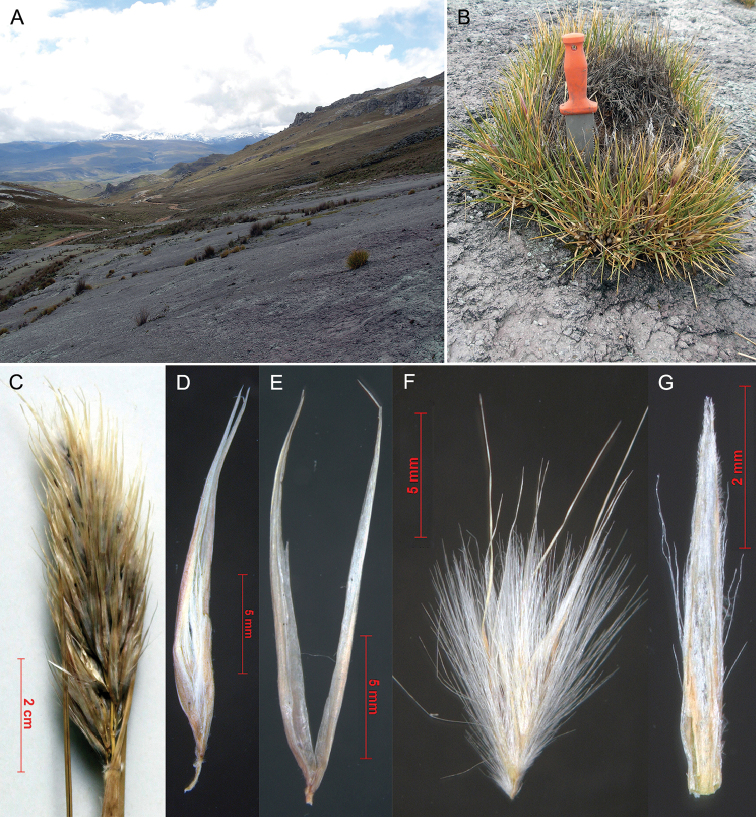
*Cortaderia echinata* (all from Peterson 21587). **A** habitat on bare rock slabs **B** habit, forming a vegetable hedgehog **C** inflorescence **D** spikelet, somewhat squashed (all very compact in the inflorescence) **E** glumes **F** floret package, with three florets, note long lemma indumentum **G** palea with sparse indumentum on the lateral palea flaps. **A** and **B** were photographed by Paul Peterson and Robert Soreng.

####### Conservation status.

Known only from the type collection.

####### Phenology.

Flowering month March or April.

####### Taxonomy.

The small compact hedgehog form with pungent leaves is similar to *Cortaderia pungens*, from which it differs by the shattering leaves and the longer spikelets (glumes 15–25 mm long). The shattering leaf-sheaths link the species to *Cortaderia boliviensis*, but it differs by the very different growth form. The compact inflorescences are reminiscent of *Cortaderia egmontiana*, but the pungent leaves provide a simple diagnostic difference.

The leaf anatomy is reminiscent of that of *Cortaderia bifida*, but the outer bundle sheath is not lignified, and form an extension adaxially on the vascular bundles, connecting them to the lignified anchor-shaped girders.

##### Bifida group

The leaf sheaths of this group are highly lacerated and form a tangled mat around the base of the plant. Anatomically there is nothing unusual about these species. The distinction between *Cortaderia columbiana* and *Cortaderia roraimensis* needs critical investigation.

###### 13. Cortaderia bifida

Taxon classificationPlantaePoalesPoaceae

Pilg., Bot. Jahrb. Syst. 37: 374. 1906.

Figs 1J , 3H

Cortaderia bifida Pilg., Bot. Jahrb. Syst. 37: 374. 1906. Type: Peru, “zwischen den Tambo Yuncacoya und Ramospata (Weg von Sandia nach Chunchusmayo), 2000–2400m”, 27 Jul. 1902, A. Weberbauer 1328 (lectotype, designated as holotype by Connor & Edgar, Taxon 23: 597 (1974): B-100217561! (http://ww2.bgbm.org/Herbarium/specimen.cfm?Barcode=B100217561); isolectotype: US!).
Cortaderia aristata Pilg. Bot. Jahrb. Syst. 37: 375. 1906. Type: Peru, Prov. Huamalies, Dep. Huanuco, “Berge südwestlich von Monzon, 3400–3500m”, 11 Jul. 1903., A. Weberbauer 3349 (lectotype, designated as holotype by Connor & Edgar, Taxon 23: 597 (1974): B-100217562! (http://ww2.bgbm.org/Herbarium/specimen.cfm?Barcode=B100217562); isolectotypes: K!, US!).
Cortaderia trianae Stapf ex Conert, Syst. Anat. Arundineae 100. 1961. Type: New Granada, February 1892, J. Triana 289 (lectotype, designated as holotype by Conert, Syst. Anat. Arundineae 100 (1961): K!).

####### Etymology.

*bis* (Latin) = twice + *fidu*, divide, this presumably refers to the lemma setae.

####### Taxonomy.

This species can be diagnosed by the combination of the lacerated sheath bases, the long awns and especially the long setae. The shape of the lemmas with lobes and setae are shared with *Cortaderia hapalotricha*, and the curly fibrous leaf remains are similar to *Cortaderia roraimensis*. It is separated from *Cortaderia roraimensis* by the hairy lemmas and by the much longer awns and setae. It differs from *Cortaderia hapalotricha* by the glabrous adaxial surface above the ligule and the scaberulous inflorescence branches. From the other tall species, *Cortaderia nitida*, it can be separated by the longer awns (more than 8 mm long). The long awns and setae result in the inflorescences looking similar to those of *Cortaderia peruviana*, but the bases of the plants are quite different. Consequently, it can be difficult to determine collections which consist only of inflorescences.

The leaf anatomy (Fig. 3H), in transverse section, shows shallow abaxial groves and deep adaxial clefts. Adaxially there appear to be no papillae (different from the *Cortaderia hapalotricha* anatomy). Abaxially below the epidermis are large colourless cells. The outer bundle sheath of the primary vascular bundles are completely lignified. Thus broadly similar to the *Cortaderia halalotricha* anatomy, but differing in a number of traits.

###### 14. Cortaderia planifolia

Taxon classificationPlantaePoalesPoaceae

Swallen, Contr. U.S. Natl. Herb. 29: 253. 1948.

####### Type.

Colombia, Dept. Valle del Cauca, Cordillera Occidental, extremo N, vertiente NW, entre Alto del Buey y Quebrada de los Ramos, 12 Oct. 1944, J. Cuatrecasas 18059 (lectotype, designated as holotype by Connor & Edgar, Taxon 23: 601 (1974): US 00133442!).

####### Etymology.

*planus* (Latin) = flat + *folium* (Latin) = leaf. Leaf-blades flat.

####### Taxonomy.

*Cortaderia planifolia* has many similarities to *Cortaderia pungens*, but is separated by the flat or folded, but not rolled, leaves; somewhat taller tussocks (05-1 m, compared to 0.2-0.5m); adaxial leaf surface above the ligule glabrous; glumes 8-15 mm long, compared to 12-16 mm; lemmas 4-8 mm, compared to 3-4 mm long; lemma awn less than 8 mm long, compared to more than 9 mm in *Cortaderia pungens*. These numerous small differences suggest that these are two species.

It has also been grouped with *Cortaderia hapalotricha*, from which it differs by the smaller size, the flat leaves glabrous above the ligule, the shorter lemma awn and setae.

Leaf anatomy not investigated.

###### 15. Cortaderia hapalotricha

Taxon classificationPlantaePoalesPoaceae

(Pilg.) Conert, Syst. Anat. Arundineae 102. 1961.

Figs 1K , 3I

Danthonia hapalotricha Pilg., Bot. Jahrb. Syst. 25: 715. 1898. Type: Colombia, Páramo between Usme and Pasca, Cudinamarca, June 1868, M. A. Stübel 111C (lectotype, designated as holotype by Connor & Edgar, Taxon 23: 598 (1974): B, frag. US!).
Cortaderia scabriflora Swallen, Contr. U.S. Natl. Herb. 29: 252. 1948. Type: Ecuador, near Toreador, between Molleturo and Quinoas, Province of Azuay, along lake shore, 15 June 1943, J. A. Steyermark 53188 (lectotype, designated as holotype by Connor & Edgar, Taxon 23: 602 (1974): US 00027057!; isolectotype: NY!).

####### Etymology.

*hapalos* (Greek) = soft + *thrix* (Greek) = hair. It presumably refers to the densely pubescent rhachilla.

####### Nomenclatural comments.

The type specimen of *Cortaderia scabriflora* is intermediate between *Cortaderia hapalotricha*, *Cortaderia pungens* and *Cortaderia planifolia*. It has the lemma structure of *Cortaderia pungens*, the folded leaves typical of *Cortaderia planifolia*, the pungent leaves typical of both, but the size of *Cortaderia hapalotricha*. Overall, it approaches *Cortaderia hapalotricha*.

####### Taxonomy.

Connor and Edgar (1974) note “The golden brown panicles with very hairy branches are obvious characteristics of this species.”, but these characters are variable in the species. *Cortaderia hapalotricha* is morphologically very close to *Cortaderia columbiana*, especially by the inner leaf surfaces directly above the ligule being densely and finely woolly. Genetically, the two species are strongly supported as sister species. *Cortaderia hapalotricha* can be separated from *Cortaderia columbiana* by the longer glumes, which are much longer than the spikelets, by the denser inflorescences, and by the lemmas which have well developed setae. It is also similar to *Cortaderia bifida*, but the lemmas are longer and the setae shorter. Most convincing might be anatomical differences, these need to be corroborated with more sections. The leaf anatomy and spikelet structure indicate a very close relationship with *Cortaderia pungens*, and the two might just be ecological variants of each other. However, the growth form is quite different, and we keep them separate on this basis.

Leaf anatomically (Fig. 3I) this species is very similar to *Cortaderia columbiana*, with well developed adaxial ribs, and girders linking the vascular bundles to both surfaces, as well as well developed adaxial epidermal papillae. The only difference may be the absent or poorly developed abaxial subepidermal sclerenchyma layer.

###### 16. Cortaderia columbiana

Taxon classificationPlantaePoalesPoaceae

(Pilg.) Pilg., Bot. Jahrb. Syst. 37 (Beibl. 85): 65. 1906 .

Fig. 1L

Gynerium columbianum Pilg., Bot. Jahrb. Syst. 27: 31. 1899. Type: Colombia, Merida, sine data, J. W. K. Moritz 1558 & 1559 (lectotype, designated by Connor & Edgar, Taxon 23: 597 (1974): B 10 0217508! (http://ww2.bgbm.org/Herbarium/specimen.cfm?Barcode=B100217508); isotype: US! frag. ex B). Note: The other sheet collected by Moritz (B 10 027507) is Cortaderia hapalotricha (Connor and Edgar 1974).
Cortaderia parviflora Swallen, Contr. U.S. Natl. Herb. 29: 253. 1948. Type: Venezuela, between La Trampa and Casadero, State of Merida, 28 April 1944, J. A. Steyermark 56182 (lectotype, designated as holotype by Connor & Edgar, Taxon 23: 600 (1974): US 00133441!).

####### Etymology.

-*ana*, indicating connection. From Republic of Colombia.

####### Taxonomy.

Connor & Edgar (1974) imply a similarity to *Cortaderia hapalotricha*, but note that the panicle is longer, more laxly flowered, and dull brown, and that this separates the two species. *Cortaderia columbiana* is superficially similar to *Cortaderia hapalotricha*, and also has short felty hair on upper leaf surface above the ligule, but is different by the shorter setae. Leaf anatomically they can be separated by the presence of a continuous lignified sub-epidermal layer on the abaxial side. It is also very similar to *Cortaderia roraimensis* by the lemma shape, in particular with the very short setae. However, the plant bases differ: in *Cortaderia roraimensis* the leaf bases are lacerated and curly, a feature less well developed in *Cortaderia columbiana*. Possibly the best way to separate the two species might be by the much more villous leaf margins, and often the villous adaxial leaf surface of *Cortaderia columbiana*. Geographically, the two species are also adjacent.

The leaf anatomy is like that of *Cortaderia hapalotricha*, but differs by a continuous sclerenchyma layer below the abaxial epidermis.

###### 17. Cortaderia roraimensis

Taxon classificationPlantaePoalesPoaceae

(N.E.Br.) Pilg., Notizbl. Bot. Gart. Berlin-Dahlem 6: 112. 1914.

Fig. 3J

Arundo roraimensis N.E.Br., Trans. Linn. Soc. London, Bot. ser. 2, 6: 74. 1901. Type: British Guiana, summit Mt. Roraima, autumn 1898, F. V. McConnel & J. J. Quelch 673 (lectotype, designated as holotype by Connor & Edgar, Taxon 23: 601 (1974): K!).

####### Etymology.

-*ensis* (Latin), denoting place of origin. From Mt Roraima, Guyana.

####### Taxonomy.

This is the only *Cortaderia* species from the tepuis. It is very similar to *Cortaderia columbiana*. It shares with *Cortaderia columbiana* and *Cortaderia bifida* a base of dense clustered lacerated sheaths. From the similar *Cortaderia columbiana* it is separated by the almost absent indumentum on the leaf margin directly above the simple ligule. From *Cortaderia bifida* it is distinct by the lobed lemma, where the lobes are not extended into slender setae.

The leaf anatomy (Fig. 3J) follows the same basic plan as that of *Cortaderia hapalotricha*.

## Supplementary Material

XML Treatment for Cortaderia

XML Treatment for Cortaderia selloana

XML Treatment for Cortaderia selloana subsp. selloana

XML Treatment for Cortaderia selloana subsp. jubata

XML Treatment for Cortaderia araucana

XML Treatment for Cortaderia speciosa

XML Treatment for Cortaderia hieronymi

XML Treatment for Cortaderia egmontiana

XML Treatment for Cortaderia modesta

XML Treatment for Cortaderia vaginata

XML Treatment for Cortaderia nitida

XML Treatment for Cortaderia boliviensis

XML Treatment for Cortaderia sericantha

XML Treatment for Cortaderia pungens

XML Treatment for Cortaderia echinata

XML Treatment for Cortaderia bifida

XML Treatment for Cortaderia planifolia

XML Treatment for Cortaderia hapalotricha

XML Treatment for Cortaderia columbiana

XML Treatment for Cortaderia roraimensis
